# Predicting Genetic Relatedness from Low-Coverage Sequencing Data of Human and Animal Genomes Using Various Algorithms

**DOI:** 10.3390/genes16121513

**Published:** 2025-12-17

**Authors:** Xinyi Lin, Shuang Han, Qifan Sun, Yuting Lei, Zhen Liu, Xueling Ou

**Affiliations:** 1 Faculty of Forensic Medicine, Zhongshan School of Medicine, Sun Yat-Sen University, Guangzhou 510080, China; linxy225@mail2.sysu.edu.cn (X.L.); hansh6@mail2.sysu.edu.cn (S.H.); leiyt7@mail2.sysu.edu.cn (Y.L.); liuzh278@mail2.sysu.edu.cn (Z.L.); 2 Guangdong Province Translational Forensic Medicine Engineering Technology Research Center, Sun Yat-Sen University, Guangzhou 510080, China; 3 MPS’s Key Laboratory of Forensic Genetics, National Engineering Laboratory for Crime Scene Evidence Investigation and Examination, Institute of Forensic Science, Ministry of Public Security (MPS), Beijing 100038, China; sunqifan@cifs.gov.cn

**Keywords:** low-coverage whole genome sequencing, single nucleotide polymorphism, kinship classification, human genomes, animal genomes

## Abstract

**Background/Objectives**: The further application of high-coverage whole genome sequencing in fields such as paleogenomics, forensic investigations, and conservation genomics is impeded by two major barriers: extremely high costs and stringent sample requirements. Utilizing low-coverage sequencing offers a practical solution to these constraints; however, this approach introduces a primary challenge—the necessity to reconstruct distorted genomic information for downstream analysis. **Methods**: Analytical experiments conducted on low- to medium-coverage sequencing data confirmed the accuracy of several existing methods for inferring relationships up to the third degree and distinguishing unrelated individuals. Subsequently, efforts were made to evaluate allele-frequency-independent methods within animal genomics, where analyses are likely to encounter challenges such as uncertain allele frequencies, diverse sample types, and suboptimal sample quality. Kinship inference was performed on a total of 33 pairs of animal samples across three species, comprising nine parent–offspring pairs and four full-sibling pairs. **Results**: The analysis revealed that two efficient algorithm implementations (READ and KIN) successfully identified all unrelated pairs. Notably, among the various algorithms utilized, only KIN exhibited confusion between first- and second-degree relationships when subjected to. **Conclusions**: This study has filled a critical gap in the existing literature by conducting a comprehensive evaluation of various algorithms on low-coverage sequencing data derived from authentic human and animal samples, accompanied by detailed ground truth—a vital task that has been overlooked.

## 1. Introduction

Pairwise kinship measurement is a critical topic within the field of genetics, with extensive applications that range from ancient DNA research aimed at elucidate social customs and burial cultures in historical societies, to forensic investigations that confirm unknown identities through biological samples in cases involving missing and unidentified persons. Additionally, it plays a significant role in genome-wide association studies (GWAS) where close relatives represent nuisance factors that need to be removed. To draw accurate inferences, it is essential to have sufficient biological information and effective kinship algorithms. Although classical tools for close kin identification have been microsatellites (STRs), a growing number of studies today perform relatedness estimation using genome-wide SNP data instead [1]. Recent advancements in high-throughput sequencing and SNP microarray technologies now enable researchers to obtain genome-wide SNP genotype datasets within a single experimental run. This development enhances the system’s efficacy for resolving kinship determinations using SNP-based platforms. In recent years, decreasing sequencing costs have empowered more forensic genealogists to incorporate whole genome sequencing (WGS) data into their research endeavors, which plays a crucial role in uncovering additional genetic information. Consequently, computationally efficient tools for genome-wide kinship coefficient estimation [2,3,4] and algorithms for detecting IBD segment algorithms [5,6,7,8] have emerged specifically tailored for this purpose.

When high-quality sequencing data and an appropriate background population sample for inferring haplotype frequencies are available, relatedness determination is a relatively straightforward task. However, evidential DNA samples of suboptimal quality are often encountered in various fields of biological research. In such cases, an individual may be represented by only dozens of thousands of SNPs with just a single allele per SNP due to the degraded nature of the DNA in these samples. Under these circumstances, genotype imputation can be employed to recover sufficiently reliable SNP genotypes. In our previous study [9], kinship analyses conducted on sequencing data with ~1× coverage and SNP genotype profiles from reference relatives derived from high-coverage sequencing demonstrated satisfactory accuracy for up to 5th-degree relatedness using the imputation strategy implemented in GLIMPSE [10]. However, when applying kinship analysis where both pairwise samples consist of DNA profiles with limited sequencing depth—such as human remains from the same pedigree recovered at disaster sites, trace DNA evidence extracted from different individuals at the same or different crime scenes, and low-coverage sequencing samples used in clinical screening—methods reliant on imputation face three primary limitations. First, the requirement for imputation across an increased sample size further prolongs the computationally intensive process. Second, errors from imputation may accumulate across samples, resulting in greater inaccuracies in estimating kinship coefficients or IBD segments. Finally, current imputation methods struggle to yield a sufficient number of reliable diploid genotypes with extremely low-coverage sequencing data (below 0.5×) [11].

Since ancient DNA exhibits similarities to DNA samples collected from crime or disaster scenes, several imputation-free kinship algorithms that have garnered significant attention in aDNA research are also considered applicable in forensic investigations. Most existing methodologies utilize either stochastic pseudo-haplotype calling [12,13] or genotype likelihood estimation [3,14,15,16,17,18] to address the genotype uncertainty due to limited coverage. With appropriate genomic inputs, algorithms such as maximum likelihood estimation [14], hidden Markov modelling [19] or moment estimation [17] are utilized to characterize the most likely relationship distance between genomes. A previous benchmarking exercise [20] reported that with over 10,000 SNP variants available for comparison, it is possible to identify most 1st- to 3rd-degree relatives based solely on the pairwise mismatch rate of simulated pseudo-haplotype data. Furthermore, accurate classification of 3rd-degree relatives represents the current detection boundary achievable by existing methods in the absence of population allele frequencies. Conversely, while allele frequency-based methods face limitations due to a scarcity of reference genomes and frequency data when applied to aDNA, their operational validity in forensic science is compromised by ongoing gene flow resulting from modern demographic dynamics—particularly evident in admixed populations characterized by multiple ancestral contributions and temporal admixture stratification. Nevertheless, it remains unequivocal that an increase in prior knowledge regarding allele distributions and inheritance patterns within the target population significantly enhances predictive power for resolving consanguinity. Although the applicability of the aforementioned algorithms has been demonstrated through simulation experiments and a limited number of case reports in the context of aDNA [21,22], their performance in large-scale analyses of authentic sequencing datasets remains understudied.

Dense SNP genotype data is of paramount importance not only for human genome research but also for selective breeding, wildlife conservation genetics, and the resolution of animal theft cases. In the latter two contexts, animal samples submitted for analysis may include bodily fluids, hair, feces, or other carriers that vary in quality, presenting formidable challenges for both detection and comparison. Traditional processes involving STR and SNP microarray genotyping requires species-specific genetic marker screening, primer design, specificity validation, and reagent kit development. Consequently, protocols established for one species cannot be readily applied to others. While WGS is a highly versatile and scalable technology, obtaining sufficient DNA templates from low-quality samples remains a significant hurdle that often limits the generation of high-coverage sequencing data. More critically, both captive and companion animals are influenced by domestication effects and artificial directional selection; this results in varying allele frequencies associated with phenotypic or behavioral traits across different breeds [23,24]. It is evident that the available population genetic databases accessible to specialists engaged in practical work are severely inadequate. Currently, only a limited number of published studies have sought to leverage dimensionality-reduced clustering based on genome-wide information for animal lineage classification [25]; however, no definitive kinship determinations have been made that could lead to persuasive conclusions in forensic practice. In contrast, integrating imputation-free kinship algorithms with genome-wide data presents a more promising strategy for developing protocols that can be easily adapted for analyses across multiple species while providing comprehensive kinship assessments.

This study aims to evaluate the efficacy of various imputation-free algorithms based on distinct principles for kinship analysis, utilizing downsampled WGS data with insufficient coverage from a southern Chinese Han cohort and 15 non-human samples (human: 1×, 3×, 6×; cattle, canine and domestic cat: 0.5×, 1×, 10×). To our knowledge, this represents the first investigation to apply such methodologies across multiple species. Through a comprehensive evaluation, we explored the impact of sequencing depth and SNP ascertainment on relationship determination. This exploration revealed critical factors essential for establishing relevant forensic identification standards. The primary performance metrics—coefficient estimation precision and discriminative capacity among kinship categories—are presented in detail in the following sections.

## 2. Materials and Methods

### 2.1. Data and Sample Collection

Private high-coverage WGS data were obtained from our previous publication [9], which included 30 and 56 individuals belonging to two well-documented southern Chinese Han pedigrees, each with a fully established genealogy. The complete pedigree structure schematic and comprehensive methodological details can be consulted in the Figure S1 of the prior publication [9]. All procedures were conducted with informed consent and in accordance with the ethical standards of the Human Subjects Committee at Zhongshan School of Medicine, Sun Yat-Sen University (No. [2020]018), the 1964 Helsinki Declaration and its later amendments or comparable ethical standards.

In addition, we collected a total of 15 biological samples, including whole blood and saliva stains, from six well-documented animal pedigrees sourced from veterinary clinics and farms. These samples encompassed three types of animals: domestic cats, dogs, and cattle. Each type represented two distinct pedigrees, resulting in 2 pairs of parent–offspring relationships for cattle, 2 pairs for canine, 5 pairs for cats, and four pairs of full-sibling cats (Table S1).

### 2.2. Whole Genome Sequencing

For the collected animal samples, blood-derived DNA was extracted using the Magnetic Universal Genomic DNA kit (TIANGEN, Beijing, China) on the Kingfisher (Thermofisher, Waltham, WA, USA), in accordance with the manufacturer’s recommended protocol. For the saliva stain samples, genomic DNA was extracted using the TIANamp Micro DNA Kit (TIANGEN, Beijing, China). The integrity and contamination of the extracted DNA were assessed via agarose gel electrophoresis. Total DNA quantification was performed using Qubit (Invitrogen, Carlsbad, CA, USA). During library preparation with the BGI Optimal DNA Library Prep Kit (BGI, Shenzhen, China), a specific amount of genomic DNA was fragmented; subsequently, size selection of the resulting products was conducted using magnetic beads. The size-selected DNA fragments underwent sequentially modifications including end repair, A-tailing and adaptor ligation. Following this process, amplification of the library was achieved through PCR reactions accompanied by quality control measures. Next, the double-strand library was denatured and circularized to produce single-strand circularized DNA. The final synthesis of DNA nanoballs (DNBs) occurred via phi29-mediated rolling circle amplification (RCA), each containing approximately 300 copies of the original single-stranded library molecule. Ultimately, DNBs were loaded into a patterned nanoarray where sequencing reads of PE100 bases were generated with MGIseq2000 sequencing platform (BGI, Shenzhen, China).

The quality of the raw sequencing data for each sample was evaluated using FastQC software v0.11.9 (https://www.bioinformatics.babraham.ac.uk/projects/fastqc/, accessed on 22 June 2023). Following quality control measures, the filtered data were processed using BWA software v0.7.17 [26], aligning reads to the reference genome sequences of Canis familiaris (NCBI CanFam3.1), Bos taurus (NCBI Bos taurus ARSUCD2.0), and Felis_catus 9.0 (GCA 000181335.4) to generate corresponding SAM files for different types of animal samples. The SAM files were subsequently sorted and converted into BAM files utilizing samtools software v1.9 [27]. Sentieon v201911 [28] was then employed to mark duplicate sequences and perform recalibration of base quality score (QS). Finally, detection of single nucleotide variants (SNVs) for each individual was carried out using Sentieon’s Haplotyper v202308.02 [29].

### 2.3. Downsampling

To simulate the relationship determination between pairs of poor-quality samples, QC-filtered high-coverage WGS data from all 86 human participants, along with 15 animal samples were downsampled using DownsampleSam v4.1.5. This tool randomly discards a certain proportion of reads according to the retention probability (defined by the --PROBABILITY parameter), resulting in output characteristics akin to low-coverage sequencing. In this study, three datasets with progressively increasing coverages for human samples were generated by configuring the PROBABILITY parameter to 0.25, 0.1 and 0.3, respectively, this parameter was specially set to 0.08 and 0.04. Subsequently, a custom Linux script was employed to calculate the new average sequencing depth for each output dataset.

### 2.4. Imputation-Free Kinship Inference

Prior to conducting kinship inference, we combined two datasets from the 1000 Genomes Project [30]—specifically, CHB and CHS—which exclusively include samples from Beijing and southern China, in order to estimate allele frequencies. For three additional animal species, population frequency data were sourced from a genetic variant identification project involving Dengchuan cattle [31] and the Animal-SNPAtlas database [32] for variant screening purposes. A filtering step was implemented to remove rare variants (MAF < 0.01) and non-biallelic SNPs prior to subsequent downstream analyses.

To address the challenges associated with extremely low-coverage sequencing, such as sparse marker sites and unreliable genotype calls, several imputation-free algorithms [13,14,15,19,33] have been developed based on various genomic similarity measures. These methods can be categorized into two groups, depending on whether allele frequency data is required. Ultimately, three tools that depend on allele frequency and two that are independent of it were selected for evaluation in the main experiment. Additionally, frequency files (.freq) were prepared to ensure compatibility with the kinship analysis tools reliant on allele frequencies.

#### 2.4.1. Estimation of Pairwise Mismatch Rate for Pseudo-Haploid Data

For each individual, pseudo-haploid genotypes were randomly generated using ANGSD v0.920 [34] to initiate the analyses. The resulting haploid data were subsequently converted to PLINK format using plink v1.9.0 [4] and the accompanying tool haploToPlink for the pairwise mismatch rate estimator, READv2 [13], as required. The primary indicator for differentiating kinship degrees in this approach is the normalized P0, which is defined as follows.

$${P0}_{normalized}=1-\frac{P0}{{P0}_{median}}$$

READv2.0 allows users to specify a median mismatch rate derived from extensive genomic data of unrelated individuals with similar hereditary backgrounds to those of the test individuals. If not specified, the program defaults to using the median P0 estimated from the input samples for normalization. However, given that over 50% of all analyzed target individual pairs were unrelated, employing default values offers a more efficient procedure while ensuring negligible bias as reported by the developers.

#### 2.4.2. Shared IBD State Prediction Through the Best-Fit Hidden Markov Model

This: method aims to fit an optimal Hidden Markov Model (HMM) for analyzing IBD sharing between pairs of individuals. This model is designed to represent the shared IBD state along two genomes and to identify the most probable hypothesis by comparing the best-fit model with ideal IBD sharing patterns across various relationships, thereby maximizing likelihood estimates. The core algorithm is implemented in the KIN v3.1.2 Python package [19], while another Python package, KINgaroo, facilitates the direct pre-processing for all target genome alignment files (.bam). It is worth noting that normalized P0 is also invoked as a component of model parameters within KIN when conducting relatedness inferences. In line with the rationale presented in the preceding section, we employed default parameters as specified throughout this experimental phase.

#### 2.4.3. R0, R1 and KING-Robust Kinship Statistics Estimated by SFS-Based Approach

**Figure 1:**
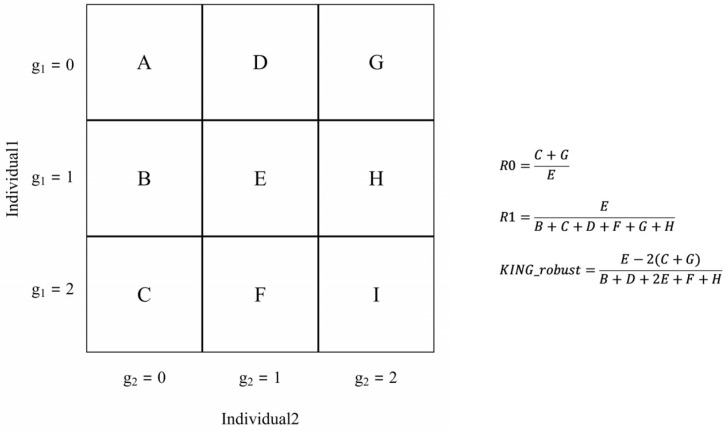
Schematic diagram of real-SFS approaches.

The genome-wide pattern of identity-by-state (IBS) sharing pairs of individuals can be succinctly characterized using three dimensions, R0, R1 and KING-robust (Figure 1). The three statistics are originally defined as follows: (1) the proportions of genomic regions in which two individuals share either 0 or 1 allele that is IBD, and (2) kinship coefficients conceptualized through the formulation $\theta =\;\frac{k_{1}}{4}+\frac{k_{2}}{2}$. For sequence data with low coverage, reformulating these statistics in terms of a two-dimensional site frequency spectrum (SFS) across target pairs has been reported to significantly enhance relatedness determination [33].

For simulated populations with large sample sizes in the earlier study, individual pairs exhibiting the same relationship appeared to cluster effectively around the theoretical values within the cubic space defined by these three statistics. Nevertheless, since the estimated two-dimensional site frequency spectrum cannot precisely capture the true distribution of allele sharing, determining a critical value that maximizes discriminatory power requires extensive testing using a substantial number of real-world samples. To ensure comparability in kinship estimation, KING-robust was employed as the primary classification criterion for this investigation due to its established reliability as a measure of relatedness. The output generated by NgsRelate [14] includes these estimates, with actual calculation performed using the realSFS method implemented in ANGSD, which will be elaborated upon in detail in the following section.

#### 2.4.4. Relatedness Estimation Based on 9 Condensed Jacquard Coefficients

NgsRelate v2.0 [14] serves as a computational platform for estimating the probabilities of nine distinct possible IBD sharing patterns, represented by condensed Jacquard coefficients, between individual pairs using genotype likelihoods. The 9 Jacquard coefficients can be linearly combined to derive various relatedness measures, with the most recognized being $\theta_{ab}=J_{1}+\frac{J_{3}+J_{5}+J_{7}}{2}+\frac{J_{8}}{4}$ and its simplified form under the assumption of no inbreeding $\theta =\;\frac{k_{1}}{4}+\frac{k_{2}}{2}$. In this study, ANGSD was configured with the -GL 2 option to produce genotype likelihood files for all downsampled samples, which were subsequently utilized as direct input for downstream estimation in NgsRelate. Despite our comprehensive pedigree analysis indicating that no inbreeding individuals were included in our sample set, we opted not to use the -o option to treat 7 Jacquard coefficients as zero due to significantly overestimated $k_{2}$ values observed among unrelated pairs during preliminary experiments. Given NgsRelate’s tendency to underestimate relatedness coefficients, we adopted two classification criteria corresponding to different kinship ranges. Firstly, we considered the theoretical lower bound of θ for 4th-degree relatives as a threshold distinguishing related from non-related individuals. Upon identifying related individual pairs, we calculated Euclidean distances from their coordinates ($k_{0}$, $k_{1}$, $k_{2}$) to theoretical reference points representing parent–offspring, full siblings, 2nd-degree relatives, and 3rd-degree relatives; these distances were then employed for kinship classification. It is important to note that accurately estimating overall IBD necessitates the assumption that variant sites are independent of one another. Consequently, this method is particularly sensitive to genetic linkage and linkage disequilibrium effects. To address this concern, we further refined our variant set by ensuring a minimum inter-marker distance of 2000 base pairs in accordance with recommendations from a prior methodological study [18].

#### 2.4.5. Likelihood Ratio Calculation Under Diverse Pedigree Hypotheses

The final component focuses on assessing the performance of the likelihood ratio method, which incorporates assumptions regarding pedigree structure when applied to low-coverage sequencing data [15]. For applications involving uncertain genotypes, a three-tiered strategy has been proposed that includes a population model, an inheritance model and an observational level model. This approach addresses issues at various levels such as population genetic structure, distinct IBD patterns and unreliable diploid genotypes. The R script lcNGS.R was downloaded from https://familias.name/lcNGS/, accessed on 12 July 2024, where only five types of relationships (Unrelated/Full Siblings/Half Siblings/First cousins/Second cousins) were defined in the original code. To enhance inference accuracy, two additional relationships (Uncle-nephew/First cousin once removed) were appended to the list of the hypotheses. Unfortunately, the validation of lcNGS’s accuracy was limited to 10 human individual pairs per relationship category and two specific sets of autosomal SNP markers derived from the ForenSeq^®^ Kintelligence Kit [35] as well as the 25K SNP capture panel [22]. This limitation arose due to the exponential increase in computational resource requirements associated with simulating inheritance process for larger numbers of genetic markers. Furthermore, this strategy contributed to mitigating the adverse effects of linkage disequilibrium on conditional probability estimates. All samples selected through stratified random sampling were submitted to the program following genotype likelihood estimation and genotype calls using BCFtools v1.16, along with the genetic map and allele frequency file created from CHB and CHS datasets.

### 2.5. Relationship Determination Using SNP Genotypes Under Permissive Threshold Criteria

In contrast to the well-defined pedigree structures observed in humans and domestic cats, the actual relationships between 4 pairs of cattle or canines remain ambiguous. Therein, we conducted a simultaneous analysis of the original sequencing data (10× coverage) from these samples employing both imputation-free methods and the traditional likelihood ratio approach—an integration of diploid genotype information with population frequency data—to identify mutually corroborating genetic evidence. Unlinked SNPs recognized by the International Society for Animal Genetics (ISAG) Standing Committees with a minimum 5× sequencing depth across all samples were selected for genotype calling. The classical R package Familias v2.4 [36] was utilized to compute likelihood ratio and confirm relatedness between animal individuals. The resulting relationships will be presented and compared against the original records as well as the inferences derived from READ and KIN.

### 2.6. Data Statistics

Statistical analyses of outputs generated by the aforementioned software packages were implemented using R programming language version 4.0.0. We primarily focus on three metrics in our evaluation: accuracy, false positive rate and false negative rate. Accuracy is calculated as the proportion of individual pairs for which the inference aligns with ground truth relative to the total pairs subjected to inference. The false positive rate is defined as the proportion of pairs of unrelated individual pairs that are incorrectly classified as close relatives (up to the 3rd degree). Conversely, the false negative rate is defined as the proportion of true close relatives that are incorrectly classified as unrelated. Figures illustrating result were primarily created using the ggplot2 library version 3.5.1.

## 3. Results

### 3.1. Downsampled WGS Data

As presented in Table 1, three downsampling products with low- or medium-coverage were sequentially generated for each human sample. Each dataset comprised 1399 sample pairs, including 120 1st-degree relative pairs, 160 2nd-degree relative pairs, 188 3rd-degree relative pairs, and 931 negative control pairs with no genetic relatedness. For the 3 non-human species examined in this study, sequencing data with approximately 10×, 1× and 0.5× coverage were prepared for subsequent kinship analyses. The following investigations will focus exclusively on the 9 datasets outlined in Table 1; however, original sequencing data from 15 non-human individuals will be utilized for further confirmation of relationship confirmation.

**Table 1:** Coverage statistics for downsampled sequencing datasets in the present study.

Dataset	Coverage (×) Mean (min-max)			
	Mid	Low-to-Mid	Low	Ex-Low
Huamn (*n* = 80)	6.845 (4.445–10.867)	3.423 (2.222–5.434)	0.856 (0.556–1.359)	
Bos taurus (*n* = 4)	9.070 (8.510–9.365)		0.726 (0.681–0.749)	0.363 (0.341–0.374)
Canis lupus familiaris (*n* = 4)	10.688 (9.223–12.220)		0.855 (0.738–0.978)	0.428 (0.369–0.489)
Felis catus (*n* = 7)	10.958 (9.094–11.893)		0.877 (0.727–0.951)	0.438 (0.364–0.476)

### 3.2. Kinship Inference on Low-Coverage Human Genomes Without Imputation

**Figure 2:**
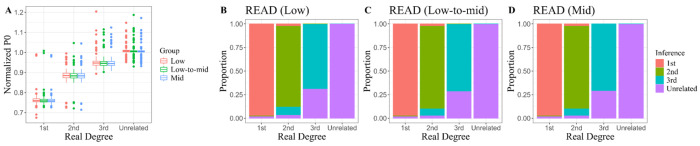
Performance of READ in kinship determination. (**A**) Distribution of Normalized P0 estimated by READ across different degrees of relatedness and non-unrelatedness. (**B**–**D**) Inferences made by READ within close relatives and between unrelated individuals using sequencing data with varied coverages.

Due to the high processing speed of the algorithms, a comprehensive analysis of all 1399 sample pairs was conducted in a single run, excluding the last method (lcNGS). This section will present the distributional characteristics of various classification indicators among distinct kinship groups as well as infer kinship degrees; these insights will further elucidate the principles underlying genealogy recognition. The simplest and most efficient approach is pairwise mismatching rate comparison, which involves random pseudo-haplotype calling performed only on the sequencing reads. The Python script READv2.py facilitates rapid estimation of normalized P0 even when handling large volumes of pseudo-haplotypes recorded in PLINK format. As indicated in the results, READv2 produced consistent estimates across three datasets with varying coverage (Figure 2B–D). While identification of 1st-degree relatives achieved an accuracy exceeding 95%, 2nd-degree relative identification maintained a modest false positive rate below 5%. However, classification reliability significantly decreased for 3rd-degree relatives, with over 25% of pairs erroneously classified as unrelated. Moreover, in some rare instances, unrelated individual pairs were incorrectly identified as 3rd-degree relatives, indicating a greater confusion between 3rd-degree relatedness and non-relatedness. These observations could be correlated to the non-linear increase in mean normalized P0 with genetic distance shown in Figure 2A.

**Figure 3:**
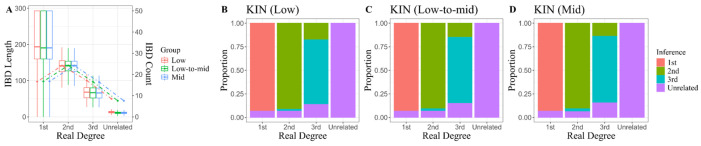
Performance of KIN in kinship determination. (**A**) The distribution of IBD length and IBD count identified by KIN across various degrees of relatedness and unrelatedness. (**B**–**D**) Inferences drawn by KIN within close relatives and between unrelated individual pairs using sequencing data with varying coverage levels.

Notwithstanding a slight increase in analysis time, the multi-feature modelling method is expected to capture more advantageous genetic information for kinship identification. IBD-based approaches demonstrate an enhanced capacity for discerning kinship relationships. However, these methods are seldom employed when analyzing low-coverage data due to their vulnerability to significant genotype miscalls. The kinship inference module KIN provides an IBD segment detection strategy specifically tailored for low-coverage sequencing data, addressing some of the aforementioned challenges. During intergenerational inheritance, homologous DNA segments undergo repeated separation and truncation due to recombination until they fall below the detection threshold or cease transmitted to subsequent generations. In theory, with an increasing number of meiotic divisions, the total length of IBD segments is expected to progressively decrease while their count initially rises before eventually declining. (Note that in parent–offspring pairs, the entire genome is considered a single IBD segment.) As illustrated in Figure 3A, KIN consistently reproduced these f characteristics across samples with low to moderate sequencing coverage. Furthermore, KIN achieved slightly higher accuracy (>68.6%) and significantly lower false positive rate (<13.8%) for 3rd-degree relatives compared to the previous P0-based method (Figure 3B–D). Additionally, it enhances the precision with which 2nd-degree relatives can be identified; this improvement is evidenced by fewer instances where 2nd-degree relatives were misclassified as 3rd-degree relatives (<2%), and no confusion arose between 1st- and 2nd-degree relatives. One notable limitation was the abnormal increase in false positive rates among 1st- and 2nd-degree relatives (~7%). IBD segment detection indicated that the IBD lengths of these individual pairs were considerably shorter than average, reflecting imprecise recognition of IBD segments.

**Figure 4:**
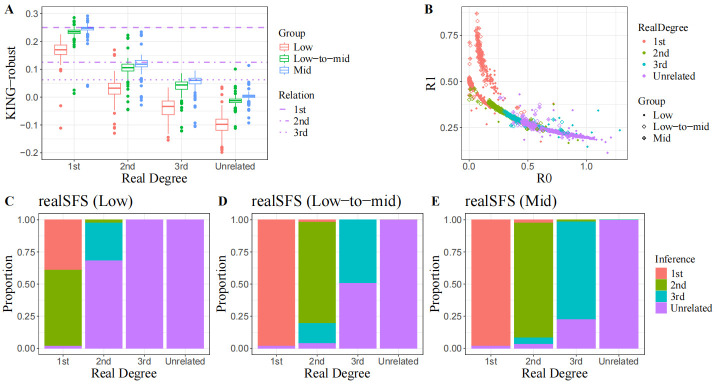
Performance of real-SFS in kinship determination. (**A**,**B**) The distribution of KING-robust, R0, and R1 estimates generated by real-SFS across various degrees of relatedness and unrelatedness. (**C**–**E**) Inferences drawn by real-SFS for close relatives as well as between unrelated individuals utilizing sequencing data of varying coverage levels.

The real-SFS method proposed by Ryan K. W. et al. represents the third imputation-free approach validated in this study, which does not require the provision of population allele frequencies. The KING-robust parameter estimation revealed a notable deficiency: as sequencing coverage decreased, real-SFS tended to produce underestimates (Figure 4A). For instance, at an average sequencing depth of approximately 1×, real-SFS yielded an average KING-robust estimate of 0.165 for 1st-degree relatives, falling below the theoretical lower bound. This trend renders it nearly impossible to accurately infer kinship from sequence data characterized by low quality (Figure 4C). However, both parameter estimation bias and classification errors diminished in datasets with higher coverage (Figure 4A,D,E). In the Low-to-mid dataset, real-SFS closely trailed READ in terms of performance, with only a narrow margin separating their results. As coverage increased to around 6×, real-SFS started to demonstrate potential superiority over the previous two methods. The estimated results for R0 and R1 are illustrated in Figure 4B. Across all three datasets examined, the distributions of 2nd- to 4th-degree relatives exhibited substantial overlap, with a distinct cluster of 1st-degree relatives was observed in a specific region in the upper left quadrant. We hypothesize that estimating the rational range of R0/R1 by treating it as a function of allele frequency f (where 0<f<1), parameterized by fixed theoretical values ($k_{0}$, $k_{1}$, $k_{2}$), may not be well-suited for making individual-level inferences due to considerable variation in the observed ($k_{0}$, $k_{1}$, $k_{2}$) resulting from genotyping uncertainties.

**Figure 5:**
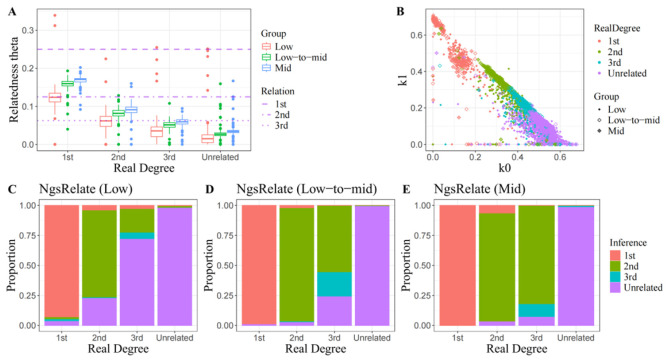
Performance of NgsRelate in kinship determination. (**A**,**B**) The distribution of relatedness coefficients θ, $k_{0}$ & $k_{1}$ estimated by NgsRelate across different degrees of relatedness and unrelatedness. (**C**–**E**) Inferences made by NgsRelate for close relatives as well as between unrelated individuals using sequencing data of varying coverages.

The following tests were conducted on two allele-frequency-dependent approaches, both of which represent genotyping uncertainty by calculating genotype likelihoods. Consistent with several previous studies, the Jacquard coefficient estimation method consistently yielded lower values than the theoretical expectations when calculating relatedness coefficients in low-coverage sequencing data (Figure 5A). Despite filtering variants based on minor allele frequency (>0.01) and intergenic distance (>2000 base pairs), maximum likelihood estimation still assigned a higher probability to those IBD states that theoretically should have a zero probability in non-inbreeding scenarios. This abnormal estimation not only caused an underestimation of key coefficients ($J_{8}$ and $J_{7}$, also known as $k_{1}$ and $k_{2}$) used for identifying related pairs but also led to an underestimation of the $J_{9}$ ($k_{0}$) coefficient among unrelated pairs. Consequently, the vast majority of unrelated pairs appear indistinguishable from 3rd-degree relatives along this single dimension (Figure 5B). Upon recognizing that classification based solely on the relatedness coefficient was unreliable, we ultimately decided to use it as a boundary between related and unrelated individuals. For individual pairs classified as related, the final kinship degree was determined by calculating the Euclidean distance from the estimated parameters ($k_{0}$, $k_{1}$, $k_{2}$) to theoretical points representing various close relatives. The classification criteria functioned effectively for 1st- and 2nd-degree relatives; however, accurate identification of 3rd-degree relatives was rarely observed in any of the three datasets (Figure 5C–E).

**Figure 6:**
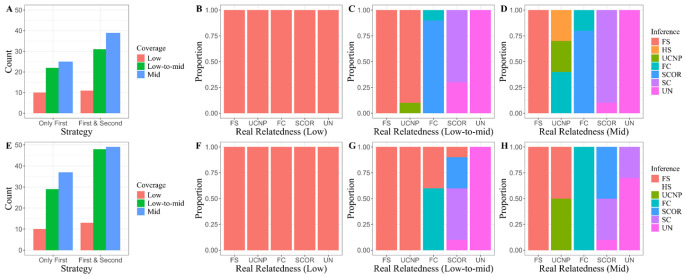
Inferences made by lcNGS across five relationship categories, encompassing 1st- to 4th-degree relationships as well as connections between unrelated individuals. (**A**) The number of cases that accurately identified the correct relatedness, considering Only the Fist hypotheses or both the First & Second hypotheses, with approximately 10,000 sites included in the ForenSeq^®^ Kintelligence Kit at varying coverages. (**B**–**D**) Inferences made by lcNGS for different kinds of relatives and between unrelated individuals using variant from the ForenSeq^®^ Kintelligence Kit at varying coverages, considering both the First & Second hypotheses. (**E**) The number of cases that accurately identified the correct relatedness when considering Only the Fist hypotheses or both the First & Second hypotheses with approximately 25,000 sites included in a 25K panel at varying coverages. (**F**–**H**) Inferences made by lcNGS for different kinds of relatives as well as between unrelated individuals using variant sites from in a 25K panel at varying coverages while considering both the First & Second hypotheses. FS: Full siblings. HS: Half siblings. UCNP: Uncle-Nephew. FC: First cousins. SCOR: Second cousins once removed. SC: Second cousins. UN: Unrelated.

In contrast to the aforementioned approaches, kinship inference in lcNGS employs hypothesis comparison rather than parameter estimation. To assess the effectiveness of this method, we randomly selected 10 individual pairs from each relationship category based on pedigree records. Given that the algorithm requires substantial computation time for probability calculations across numerous variants, initial tests were performed using sequencing data from nearly 10,000 sites targeted by the ForenSeq^®^ Kintelligence Kit (QIAGEN, Hilden, Germany) (Figure 6A–D). In the dataset with 1× coverage, the method exhibited no discriminative power regarding relationships, likely due to an insufficient number of overlapping sites to provide adequate genetic information. As the number of overlapping sites increased, lcNGS began to demonstrating its accuracy in distinguishing between relatives and non-relatives. For two specific relationship groups, uncle-nephew and first cousins, the hypothesis with the highest likelihood ratio deviated from the true relationship by only one degree of kinship, although it rarely identified the correct category. We further documented each individual pair’s relationship hypothesis corresponding to the second highest likelihood ratio and found that both first and second hypotheses in subsequent datasets enhanced the ability to identify true relationships. The following experiments employed a 25K panel comprising more variant sites to validate these findings (Figure 6E–H). Despite occasional misclassifications where unrelated individuals were incorrectly identified as second cousins, there was still a relative increase in the overall proportion of correct first inferences. Importantly, when analyzing data with moderate coverage levels, over 90% of cases ranked true relationships within the top two positions for likelihood ratios.

### 3.3. The Remarkable Capacity to Discriminate Between Distinct 1st-Degree Kinship Types

In the preceding experiment, both the pairwise mismatch rate approach (READ) and the HMM framework (KIN) for IBD sharing patterns demonstrated high and robust sensitivity in detecting 1st-degree relatives. To date, no reliable method has emerged that can accurately differentiate between various kinship types within the same degree when only low-coverage sequencing data are available, with the exception of 1st-degree relationships. Given the increasing demand to refine genetic structures within a pedigree, evaluating how effectively a method distinguishes among relatives sharing an identical kinship degree is a critical aspect of genealogical investigation and will be presented in this section. As shown in Table 2, six instances incorrectly identified by READ exhibited normalized P0 values falling within the range indicative of 1st-degree relationships; however, these cases could not be resolved into specific relationship types. With one exception classified as involving 2nd-degree relatives, the remaining two were false negatives. In terms of KIN’s inference results, eight errors were recorded—each being false negative. Since the pairwise mismatch rate also serves as a reference feature employed for classification in KIN, it is suspected that some false negatives arose from irreconcilable contradictions between IBD system assessments and normalized P0 judgement. Nevertheless, neither method caused any confusion when adjudicating between parentage verification and full-sibling identification, thereby demonstrating their stability in this regard.

### 3.4. First-Degree Kinship Inference Conducted on Low-Coverage Animal Genomic Data

Animal genome studies exhibit characteristics similar to those focusing on ancient human genomes, particularly regarding the uncertainty surrounding the background population. Drawing inspiration from this parallel, we applied two of the three frequency-independent methods that have been validated in simulated or real-world ancient human genomic data to identify kinship among animals. The medium-coverage, undownsampled sequencing data were initially analyzed as high-quality references. The kinship inferences for all 33 animal pairs closely aligned with pedigree investigations and independent results presented in the subsequent section, with the exception of three parent–offspring pairs belonging to pedigree F1 of domestic cats, for which the P0-based method READ yielded imprecise outcomes (Table 3). Although READ struggled to differentiate specific kinship relationships among these pairs within sequencing data at 1× or 0.5× coverage, its inference that they all shared a 1st-degree relationship remained robust. Notably, even when coverage decreased to 0.5×, we were pleased to observe that no misidentifications occurred in the inferences made for other animal pairs by READ. In contrast, KIN demonstrated less impressive performance in this regard. Within sequencing data at 1× coverage, KIN misidentified two out of three full-sibling pairs as parent–offspring relationships belonging to pedigree F1 of domestic cats, representing an accuracy rate virtually identical to that achieved by READ. However, KIN began to blur the distinction between 1st- and 2nd-degree relatives at lower coverage levels. For instance, regarding a cattle parent–offspring pair and an additional full-sibling pair of domestic cats misclassified by KIN, their true relationship was ranked as a secondary guess following a designation of 2nd-degree kinship due to marginally lower likelihoods. Further examination of KIN’s output revealed that even when analyzing data with a depth of 1× coverage, the evidence supporting the true kinship hypothesis remained compelling, with log-likelihood ratios of 0.105 and 1.147, respectively. A deeper underlying issue relates to the inaccurate detection of IBD segments, which is characterized by a moderate decline in total IBD length and a shift in the number of IBD segments from singular to plural. This alteration can be attributed to KIN’s inability to accurately resolve shared alleles at heterozygous loci under progressively reduced coverage, leading to erroneous truncation of IBD segments. Specific inferences and supporting information can be accessed in Supplementary Table S2.

### 3.5. Examination of Relationships in Non-Human Species Using the Classical Likelihood Ratio Approach

Following the exclusion of variants with sequencing depths below 5×, a total of 63 bovine SNPs and 201 canine SNPs were incorporated into the analysis. For bovine SNPs, population frequency data was obtained from average statistics published by ISAG, which encompassed information from 23 cattle breeds totaling 4372 individuals. In contrast, due to ISAG’s lack of publicly available population genetic data for its proposed canine SNPs, allele frequencies retrieved from the Animal-SNPAtlas database, which reflect the genetic profiles of these SNPs within Chinese canine breeds, were adopted for this analysis. Preliminary likelihood ratio computations were performed assuming the absence of mutations and genotyping errors. However, to mitigate potential systemic estimation bias arising from single-locus errors, any conditional likelihood value of zero for a given SNP was adjusted to 0.001—a figure approximating the lower limit of WGS error rates. Consequently, paternity was confirmed for four recorded pairs of cattle and canine based on genetic evidence; notably, no unrelated canine pairs exhibited a likelihood ratio greater than 1 under any related hypothesis (Table S3). Unexpectedly, four cattle pairs lacking apparent familial connections exhibited likelihood ratios greater than 1 but remained significantly below the identification threshold (>10,000) when assuming 2nd- or even 1st-degree kinship relationships, suggesting that these two families may share a recent biological relationship. While our experiments analyzing animal samples aimed to identify relatives within 3rd-degree kinship, it is imperative to remain vigilant regarding the influence of high genetic similarity on imprecise kinship identification systems in order to avoid drawing arbitrary conclusions.

## 4. Discussion

Considerable advancements have been made in both the methodology of investigative genetic genealogy since its inception and its application to fields such as genomic research and forensic science [37,38]. To address the challenges posed by inexpensive yet low-coverage WGS and poor-quality outdoor samples, frameworks like GLIMPSE [10] have been also developed for phasing and imputation using probabilistic genotypes. While acknowledging the inevitable presence of phasing and imputation errors, our previous study demonstrates that with sequencing data at approximately 1× coverage, appropriate threshold parameters can be set, allowing reasonable variant filtering can accurately distinguish most related individuals from unrelated ones [9]. This efficacy allows the application of imputation-dependent methods accross various forensic scenarios—such as identifying human remains at disaster sites or linking cold cases involving closely related suspects. However, when it comes to the rapid detection of admixed populations, racial minorities or non-human species in remote areas, developments that enable direct extraction of sequencing information present greater potential to reduce analysis time while eliminating reliance on population panels. Admittedly, the effectiveness of these methods is limited in reconstructing distant relationships; moreover, their performance in determining close relatedness has primarily been validated through existing studies using simulated genetic data [20]. One study selected authentic ancient human genome datasets as subjects to evaluate multiple methodologies but regrettably lacked conclusive genetic structures between samples for interpretation [21]. In this study, WGS data were collected from two human pedigrees along with 15 animal samples. Subsequently, downsampling processes were employed to generate data characteristics more akin to low-coverage sequencing than simulations. Various imputation-free methods based on distinct principles have been further selected for efficacy assessment. This approach aims to enhance our understanding of their applicability in kinship identification.

The results of the kinship classification demonstrate partial validity while simultaneously exposing inherent deficiencies within these methodologies across various aspects. One particularly striking method is the pairwise mismatch rate comparison, exemplified by READ. At its core, this method aims to minimize the impact of genotyping errors through random pseudo-haplotype calls and normalizes the mismatch rate based on population averages. This normalization reflects the background characteristics of the population and facilitates comparisons among different indicators. Indeed, it allows most measurements to cluster within their theoretical intervals, thereby establishing a foundation for differentiating between individual pairs with varying kinship degrees. However, similar to other methods that rely solely on relatedness coefficients or utilize the KING-robust kinship parameter as a classification metric, this approach encounters significant limitations. As generations increase, measurement distributions between relatives and unrelated individuals tend to overlap considerably, making these methods virtually inapplicable for identifying distant relatives beyond the 3rd degree. However, this limitation is not a major concern since many practical scenarios—such as forensic investigations involving missing and unidentified individuals—frequently involve close familial relationships [39]. In such instances, the primary objective is to confirm identities rather than generate investigative leads. In addition, we observed a slight increase in accuracy when employing the congener real-SFS method on sequence data with adequate coverage. Nevertheless, this method exhibits heightened sensitivity to reductions in coverage; thus it is not recommended for datasets where coverage falls below an applicable lower limit—approximately 4× as validated by its developers [33] and corroborated by our experimental observations.

The IBD-segment-based method shows theoretical promise for identifying distant relatives, as demonstrated through evaluations using high-coverage sequence data. However, the challenges in detecting IBD segments due to low coverage means its accuracy for 3rd-degree relatives remaining below 90% in this study. Conversely, this method exhibited a higher false negative rate, which could be attributed to several factors: while genotyping errors at various sites may induce only minor variations in the genome-wide mismatch rate, their occurrence within an extended IBD segment can lead the algorithm to erroneously classify the resulting truncated homozygous segments as non-IBD. Despite KIN introducing normalized P0 as a reference parameter, it appeared ineffective in influencing the primary system’s conclusions regarding false-negative relatives. Nevertheless, both READ and KIN exhibited consistent advantages—there were no significant fluctuations in their results across the three tested coverage levels. As previously noted by separate studies [19,21], both methods have shown potential for successful application to genomic data with coverage as low as 0.05×. Due to constraints on experimental conditions, we conducted further validation exclusively on animal genomic data with approximately 0.5× coverage.

As a supplement to the aforementioned work, we evaluated two additional methods that use population allele frequencies to calculate genotype likelihoods. In experiments involving human samples, the inferences drawn from NgsRelate were found to be unconvincing, which stands in mutual inconsistency with previous reports [20]. Conversely, another investigation incorporating the NgsRelate method demonstrated that algorithms based on genotype likelihood exhibit a higher minimum coverage tolerance threshold—approximately 1×—compared to those relying on pseudo-haplotype mismatch [21]. According to its manual, NgsRelate serves as a convenient analytical tool that integrates algorithms for estimating multiple genetic coefficients. Although relatedness coefficients derived from low-coverage sequencing data have been shown to be underestimated in most prior studies [20,21,40], as corroborated by our current analyses, specific classifications according to our criteria are rarely mentioned. Researchers have indicated in their studies that such underestimation may be attributable to uneven sequencing depth across the genome in low-coverage sequencing data. This discrepancy can lead to substantial bias in the distribution of estimated likelihoods among the three possible genotypes at sites represented by a limited number of reads [41]. Another more prevalent factor is that the population allele frequencies used in the analysis do not adequately capture genetic diversity among various subpopulations, resulting in amplified noise within the final coefficient estimates. It is also important to recognize that both systematic correction and stricter filtering thresholds are essential considerations, as this tool cannot independently address linkage disequilibrium between variants. Through repeated attempts, we have determined that incorporating Euclidean distance into condensed Jacquard coefficients enhances discriminatory power for 1st- to 2nd-degree relatives. This composite classification strategy warrants further systematic investigation to validate its potential for improving kinship recognition. Conversely, the likelihood ratio comparison method represented by lcNGS is more suitable for investigative purposes. By evaluating multiple hypotheses, this approach not only differentiates more distant relatives from unrelated individuals but also narrows kinship predictions to one or two specific relationships. However, it has the drawback of requiring precise population genetic maps. The developers compared its classification accuracy with that of NgsRelate through simulations involving 1000 pairs of first cousins and 1000 pairs of unrelated individuals. While lcNGS exhibited less information loss at 3× coverage, a notable decrease was observed when compared with the 10× coverage dataset [15]—a trend that aligns with the findings of this study. It is important to note that the accuracy rates reported in evaluations of likelihood ratio-based methods are intended solely for reference purposes, as the range of potential relationships in actual pedigree investigations is likely to encompass more than merely two or three specific types. By incorporating seven different relationships, this study offers a more objective analysis. Finally, while these methods are considered to leverage population genetic information more comprehensively for kinship inference, we would like to emphasize that the availability of accurate frequency data is generally overly idealistic in practice, particularly concerning admixed individuals or non-human species. Bearing this in mind, we opted to employ only the first two effective methods—READ and KIN—when dealing with animal data.

When investigating cases of animal theft, forensic analysis is typically confined to individuals suspected of being 1st-degree relatives. To this end, the study collected 9 parent–offspring pairs and 4 full-sibling pairs of animals for validation purposes, using pairs of individuals from distinct pedigrees within the same species as unrelated negative controls. Our preliminary experiments on animal genomes produced consistent findings, particularly highlighting the robustness of the pairwise mismatch rate method for analyzing data from all three species, even at extremely low coverage. READ yielded classifications that are aligned with those derived from pedigree records and genotype-based likelihood ratio methods across all four half-sibling pairs. However, it produced less refined results (First Degree, N/A) for parent–offspring pairs of domestic cats based on pedigree F1 data. In contrast, we noted a tendency for KIN to misidentify full-sibling relationships in small-scale animal genome analyses. For three half-sibling and one parent–offspring pairs misjudged by KIN at 0.5× coverage, the likelihood of the primary relationship decreased as coverage diminished, ultimately being overtaken by an alternative hypothesis. This mechanism of error tolerance could offer valuable insights into other methodologies based on parameter estimation. Once the adverse effects of low sequencing depth on estimation are recognized—typically resulting in underestimation—it becomes evident that using dynamic hypothetical competition rather than static theoretical values as classification criteria is beneficial in mitigating confounding factors. While estimating the mean P0 value for normalization using a substantial number of unrelated sample pairs could improve inferences regarding specific individual pairs, we consider this influence to be negligible. Instead, its minimal reliance on prior population background information is a significant advantage for future applications involving rare species. Specifically, it maintains a high probability of yielding accurate inferences even when genomic data are scarce (we validated the analytical feasibility using data from as few as four individuals). Finally, it is important to note that although specific frequency data were used to define the SNP inclusion threshold (MAF > 0.01), such a strategy is not strictly necessary. Less stringent site filtering may still enable the algorithm to effectively capture differences in genomic similarity between related and unrelated individuals.

The most prominent limitation inherent to this study lies in the representativeness of the sequencing data. Although low-coverage sequencing simulations, achieved by downsampling from 30× human and 10× animal WGS data, preserve the empirical origins of reads for allele determination, they fail to accurately model the stochastic artifacts characteristic of direct low-template DNA sequencing. Particularly concerning animal genomes, the scarcity of sample pairs further restricts the credibility of assessments regarding kinship inference accuracy. Before recruiting additional low-coverage sequencing samples to advance future research, we were able to generalize the application orientation for each method through the study. Methods based on a single parameter (e.g., normalized P0) and pseudo-haplotype calling that utilize simplified genetic information can highlight advantages in analyzing low-coverage sequencing data; however, when preliminary investigations indicate that individuals of interest share a 3rd-degree or more distant kinship, more complex algorithms become necessary. Recent genealogy studies [42,43] leveraging machine learning have shown promise in enhancing the precision of human relationship identification with multiple genetic metrics. This concurrently opens up new possibilities for improving kinship inference in low-quality sequencing data.

## 5. Conclusions

The discriminatory power of classical imputation-free kinship analysis tools designed for ancient DNA has been validated through their application to the low-quality genetic data typically encountered in forensic contexts. These methods not only prove advantageous for human genealogy investigations but are also applicable to non-human species, particularly in situations where unbiased frequency data is scarce. As test sample sizes increase, the strengths and weaknesses of various inferential methodologies become increasingly evident. During our research, we observed a proliferation of novel tools developed within this field. To establish a comprehensive genealogical analysis system that meets the diverse needs across species and varying levels of data quality, two promising avenues warrant exploration: developing more efficient statistical approaches derived from genetic information and scientifically integrating multiple estimates of kinship coefficient. Moving forward, we aim to expand genomic data collection to foster innovation and validate various kinship tools leveraging different algorithms, including machine learning.

## Figures and Tables

**Table 2:** Relationship inference within 1st-degree by READ and KIN for 120 human pairs.

Coverage	Predicted by READ						Predicted by KIN				
	True	1st			2nd	UN	1st			2nd	UN
		PO	FS	N/A ^1^			PO	FS	N/A ^1^		
Low	PO	60	0	3	0	0	59	0	0	0	4
	FS	0	51	3	1	2	0	53	0	0	4
Low-to -mid	PO	60	0	3	0	0	59	0	0	0	4
	FS	0	51	3	1	2	0	53	0	0	4
Mid	PO	60	0	3	0	0	59	0	0	0	4
	FS	0	51	3	1	2	0	53	0	0	4

^1^ N/A: not applicable.

**Table 3:** Accuracy of mid- and low-coverage animal kinship inference using allele-frequency-free approaches.

Species	Coverage	Predicted by READ						Predicted by KIN				
		True	1st			2nd	UN	1st			2nd	UN
			PO	FS	N/A ^1^			PO	FS	N/A ^1^		
Bos taurus	10×	PO	2	0	0	0	0	2	0	0	0	0
		UN	0	0	0	0	4	0	0	0	0	4
	1×	PO	2	0	0	0	0	2	0	0	0	0
		UN	0	0	0	0	4	0	0	0	0	4
	0.5×	PO	2	0	0	0	0	1	0	0	1	0
		UN	0	0	0	0	4	0	0	0	0	4
Canis lupus familiaris	10×	PO	2	0	0	0	0	2	0	0	0	0
		UN	0	0	0	0	4	0	0	0	0	4
	1×	PO	2	0	0	0	0	2	0	0	0	0
		UN	0	0	0	0	4	0	0	0	0	4
	0.5×	PO	2	0	0	0	0	2	0	0	0	0
		UN	0	0	0	0	4	0	0	0	0	4
Felis catus	10×	PO	2	0	3	0	0	5	0	0	0	0
		FS	0	4	0	0	0	0	4	0	0	0
		UN	0	0	0	0	12	0	0	0	0	12
	1×	PO	2	0	3	0	0	5	0	0	0	0
		FS	0	4	0	0	0	2	2	0	0	0
		UN	0	0	0	0	12	0	0	0	0	12
	0.5×	PO	2	0	3	0	0	5	0	0	0	0
		FS	0	4	0	0	0	2	1	0	1	0
		UN	0	0	0	0	12	0	0	0	0	12

^1^ N/A: not applicable.

## Data Availability

The original contributions presented in the study are included in the article and Supplementary Materials; further inquiries can be directed to the corresponding author.

## Supplementary Materials

The following supporting information can be downloaded at https://www.mdpi.com/article/10.3390/genes16121513/s1, Table S1: Detailed sample information for 15 animal individuals; Table S2: Detailed estimation performed by READ & KIN for 15 animal individuals; Table S3: Likelihood ratios corresponding to specific hypotheses versus the unrelated hypothesis.
